# Investigating the relationship between hepatitis B virus infection and postpartum depression in Chinese women: a retrospective cohort study

**DOI:** 10.3389/fpubh.2023.1214151

**Published:** 2023-11-29

**Authors:** Wei Huang, Xiaoli Wu, Zhenzhen Yao, Yingping Gu, Xin Lai, Liping Meng, Songxu Peng

**Affiliations:** ^1^Department of Postpartum Rehabilitation, Maternal and Child Health Hospital of Hunan Province, Changsha, China; ^2^Center for Reproductive Medicine, Maternal and Child Health Hospital of Hunan Province, Changsha, China; ^3^Department of Maternal and Child Health, Xiangya School of Public Health, Central South University, Changsha, China; ^4^Department of Public Health, Baoan Maternal and Child Health Hospital, Jinan University, Shenzhen, China

**Keywords:** postpartum depression, HBV, obstetric factors, EPDS, PPD

## Abstract

**Background:**

Postpartum depression (PPD) is associated with several psychological and obstetric factors. Hepatitis B virus (HBV) infection has been linked with a high risk of depression, but little is known about the relationship between maternal HBV infection and PPD. We aimed to investigate the association between HBV infection and PPD.

**Methods:**

This retrospective cohort study included 3,808 mothers who gave birth in a hospital in southern China. Self-reported Edinburgh Postnatal Depression Scale (EPDS) was used to assess PPD. Multivariate logistic regression was used to determine whether maternal HBV infection was associated with PPD risk.

**Results:**

Of the 3,808 participants, 11.9% of mothers had PPD at 6 weeks postpartum. Two hundred and seventy-eight (7.3%) and 3,530 (92.7%) were in the HBV and control groups, respectively. Women with HBV infection were more likely to test positive for PPD (14.7 vs.11.7%). The multivariate logistic regression analysis showed that HBV-infected women did not have a significantly higher incidence of PPD (OR = 1.23; 95% CI, 0.82–1.84) than those without HBV infection in the study cohort. Parity and postpartum hemorrhage were found to be associated with PPD. In addition, our study showed that e antigen positivity was not associated with PPD risk (OR = 0.56, 95% CI 0.19–1.63).

**Conclusions:**

To our knowledge, this is the first investigation of the relationship between maternal HBV infection and PPD. In a cohort of women without prior history or family history of mental illness, having HBV infection was not significantly associated with self-reporting of PPD compared to not having HBV infection.

## 1 Introduction

Hepatitis B virus (HBV) infection is a global public health issue (1–3). More than 240 million people have chronic HBV infection, which may develop into hepatitis, cirrhosis, and hepatocellular carcinoma without timely treatment (4). HBV prevalence was 7.18% in China, with approximately 93 million individuals infected with chronic HBV (5). A recent national study showed that the chronic HBV infection prevalence in Chinese pregnant women was 6.17% (6). Chronic hepatitis B is a contagious disease that transmits through blood, sexual activities, and mother-to-child transmission (MTCT). Therefore, patients with HBV infection often experience stigma and discrimination (7). People with chronic hepatitis B infection of any severity were associated with worse mental and physical health than healthy controls (8).

Postpartum depression (PPD), a common perinatal complication, affects 6.9–20% of puerperal women (9, 10), which significantly increases the morbidity and mortality of both mothers and children (11–15). Several studies have found that women who were diagnosed with PPD had an increased risk of future depression, suicide, or increased infant mortality (16–18). In addition, accumulated evidence showed that maternal PPD has deleterious effects on children's physical growth and mental development (19). Therefore, understanding the determinants of PPD and preventing its occurrence is vital.

Previous studies have demonstrated an association between other antenatal diagnoses and birth outcomes with PPD, including gestational diabetes (20), gestational hypertension (21), and preterm birth (22). The impact of maternal HBV infection on PPD is unknown. Previous study has examined the psychiatric effects of HBV infection outside of pregnancy and the puerperium (23). Researchers have suggested that comorbid depression is common in patients with HBV infection. For instance, a Vietnamese study showed that the prevalence of depressive symptoms in patients with chronic hepatitis B was 37.5% (24). Another study by Qureshi et al. reported that 58.6% patients with chronic hepatitis B had depression (25). In 2005, Atesci et al. assessed the incidence of mental disorders in 43 patients with HBV infection and 43 healthy controls, and reported that HBV-infected patients had significantly higher depression and anxiety scores than healthy controls (26). Similar result was found in the study conducted by Yilmaz et al. (27). The impact of maternal HBV infection on PPD, however, is still unknown. Hepatitis B e antigen (HBeAg) positivity has been frequently reported to be associated with MTCT of HBV (28). Therefore, we speculated that HBeAg positivity may increase the psychological burden on these mothers, resulting in a higher PPD risk.

Therefore, there is a need to evaluate the relationship between maternal HBV infection and PPD, and determine whether HBeAg positivity increases the risk of PPD among HBsAg-positive carriers. We conducted a large-scale retrospective cohort study to identify the potential association between HBV infection and PPD.

## 2 Methods

### 2.1 Study design and population

This was a retrospective cohort study performed on mothers who gave birth at the Baoan Maternity and Child Health Hospital in Shenzhen, China, from October 2018 to June 2020. During the antenatal visit period, routine screening of hepatitis B surface antigen (HBsAg) and HBeAg status using enzyme-linked immunosorbent assay (ELISA) was performed on pregnant women. Women who returned to the hospital for a routine follow-up at 42-days postpartum were asked to undergo PPD screening. For the self-reporting of PPD, 96% pregnant women accepted in PPD screening, because it is a service paid by the government. Women aged 20 years or older who underwent HBV marker testing in this hospital were included in this study. Exclusion criteria are as follows: (1) multiple pregnancy; (2) stillbirth or birth defect; (3) presence of family history of mental illness; (4) having past psychiatric disorders; (5) refusing PPD test.

The present study was approved by the Institutional Review Board of the Baoan Maternal and Child Health Hospital, Jinan University. Informed consent was exempted due to the retrospective study design.

### 2.2 Exposure assessment

The exposure variable was the HBV infection. In the current study, HBV infection was defined as HBsAg positivity (6).

### 2.3 Outcomes

The primary outcome was PPD, measured using the Edinburgh Postnatal Depression Scale (EPDS). The EPDS consists of 10 items rated between 0 and 3. The total score varies from 0 to 30; the higher the score, the greater the PPDs risk. The reliability and validity of the EPDS for screening for PPD were assessed in Chinese population and the cut-off point of 10 was chose, with sensitivity of 0.82 and specificity of 0.86 (29). In our study, a score of 10 or more was considered an indicator of PPD, which is in line with previous studies (30).

### 2.4 Assessment of other variables

Sociodemographic data, including age, education, parity, pre-pregnancy weight, and height, were obtained from participants' electronic medical records. Information on gestational diabetes mellitus (GDM), placental abruption, postpartum blood loss, mode of delivery, gestational week, fetal sex, and postnatal conditions of the infants were extracted from the medical records. Postpartum hemorrhage is defined as a loss of blood equal to or greater than 500 mL within 24 h after delivery (31), and preterm birth as a gestational age less than 37 weeks (32).

### 2.5 Statistical analysis

Descriptive statistics were used to present the general characteristics of participants according to HBsAg status, such as means and standard deviations for continuous variables and percentages (%) for categorical variables. Student's *t*-tests or chi-square tests were used to test whether the distributions of continuous or categorical variables, respectively, differed by HBV infection status. The association between maternal HBV infection and PPD was assessed by using multiple logistic regression models. A two-sided 5% level was considered statistically significant. Statistical analyses were performed using SPSS (version 25.0, Chicago, IL, USA).

## 3 Results

### 3.1 Study patients

In total, 4,187 women with HBV infection conditions were identified. But 103 mothers who had stillbirth or malformation, and 67 participants with past psychiatric diseases or a family history of mental illness were excluded from this study. Subsequently, 92 subjects with multiple pregnancies and 117 participants with incomplete data on PPD were excluded. Finally, 3,808 puerperal women were included in the analysis, of which 453 (11.9%) were considered to have PPD. According to maternal HBV infection status, 278 (7.3%) HBV-infected women were assigned to the HBV group and 3,530 (92.8%) mothers without HBV infection were assigned to the control group.

### 3.2 Participant characteristics

Table 1 presents the general characteristics, pregnancy complications, and delivery information of the two groups. Mothers with HBV infection were older and had a lower educational level. However, we did not observe significant differences in other pregnancy complications or delivery information between the two groups, such as pre-pregnancy BMI, parity, GDM, placental abruption, PHH, delivery mode, infant sex, preterm birth, or infant illness after childbirth.

**Table 1:** Basic characteristics according to HBsAg status of women.

**Characteristics**	**HBsAg positive**	**HBsAg negative**	***P*-value**
No. of participants	278	3,530	
Age at birth (years)	30.12 ± 3.87	29.52 ± 4.03	0.015
Education level			0.001
Junior middle school or less	32 (11.5)	251 (7.1)	
Senior middle school	60 (21.6)	580 (16.4)	
College or university	186 (66.9)	2,699 (76.5)	
Pre-pregnancy BMI			0.097
< 18.5	59 (21.2)	657 (18.6)	
18.5~23.9	197 (70.9)	2,450 (69.4)	
>24	22 (7.9)	423 (12.0)	
Primipara	167 (60.1)	1,913 (54.2)	0.058
Gestational diabetes mellitus	50 (18.0)	577 (16.3)	0.478
Placental abruption	40 (14.4)	488 (13.8)	0.793
Postpartum hemorrhage	10 (3.6)	89 (2.5)	0.278
Cesarean delivery	94 (33.8)	1,130 (32.0)	0.536
Male infant	128 (46.0)	1,618 (45.8)	0.947
Preterm labor	17 (6.1)	169 (4.8)	0.323
Infant illness after childbirth	21 (7.6)	217 (6.1)	0.351

BMI, body mass index; HBsAg, hepatitis B surface antigen.

### 3.3 Maternal hepatitis B virus infection and postpartum depression

The corresponding prevalence rates of PPD in the HBV and control groups were 14.7 and 11.7%, respectively. The prevalence of PPD was not significantly different between women with and without HBV infection (*P* > 0.05). Logistic regression analysis was used to evaluate the potential association between maternal HBV infection and PPD, the corresponding analysis results are presented in Table 2. The univariate analysis showed that primipara had a higher risk of PPD (OR = 1.34; 95% CI, 1.11–1.64; *P* = 0.003) compared with multipara. HBV infection (OR = 1.31; 95% CI, 0.93–1.85; *P* = 0.128) did not increase risk of developing PPD. The results of multivariate logistic regression analysis suggested that primipara (OR = 1.47; 95% CI, 1.15–1.89; *P* = 0.002) were more likely to develop PPD. In addition, mothers with postpartum hemorrhage were found to have a higher PPD risk (OR = 1.82; 95% CI, 1.02–3.24; *P* = 0.041). However, maternal HBV infection was not associated with the increased risk of PPD (OR = 1.23; 95% CI, 0.82–1.84; *P* = 0.320).

**Table 2:** Univariate and multivariate logistic regression analyses of factors related to postpartum depression.

**Characteristics**	**Univariate**		**Multivariate**	
	**OR (95%CI)**	***P*-value**	**OR (95%CI)**	***P*-value**
Age	0.98 (0.96–1.01)	0.207	1.02 (0.98–1.05)	0.355
Education level				
Junior middle school or less	Ref.		Ref.	
Senior middle school	1.19 (0.78–1.83)	0.421	1.08 (0.66–1.76)	0.760
College or university	0.99 (0.68–1.45)	0.953	0.87 (0.56–1.34)	0.525
Pre-pregnancy BMI				
< 18.5	1.16 (0.80–1.68)	0.427	1.07 (0.70–1.64)	0.752
18.5~23.9	1.09 (0.79–1.50)	0.596	1.00 (070–1.44)	1.000
>24	Ref.		Ref.	
Primipara	1.34 (1.11–1.64)	0.003	1.47 (1.15–1.89)	0.002
GDM	0.94 (0.71–1.23)	0.650	0.99 (0.73–1.33)	0.939
Placental abruption	0.78 (0.58–1.06)	0.118	0.75 (0.54–1.06)	0.106
Postpartum hemorrhage	1.56 (0.91–2.65)	0.103	1.82 (1.02–3.24)	0.041
Cesarean delivery	0.93 (0.76–1.16)	0.527	0.92 (0.72–1.18)	0.509
Male infant	0.94 (0.78–1.15)	0.567	0.89 (0.71–1.11)	0.283
Preterm labor	0.79 (0.48–1.29)	0.339	0.73 (0.41–1.31)	0.291
Infant illness after childbirth	1.32 (0.91–1.93)	0.148	1.20 (0.79–1.83)	0.385
HBV infection	1.31 (0.93–1.85)	0.128	1.23 (0.82–1.84)	0.320

OR, odds ratio; CI, confidence interval; GDM, Gestational diabetes mellitus.

### 3.4 HBeAg status and postpartum depression

We further explored the impact of HBeAg status on maternal PPD in HBsAg-positive carriers. The results of the comparative analyses showed that HBeAg-positive mothers were younger than HBeAg-negative mothers (Table 3). Multivariable logistic regression analyses showed that HBeAg positivity (OR= 0.54, 95% CI 0.18–1.58, *P* = 0.261) was not significantly associated with an increased rate of PPD compared to HBeAg negativity.

**Table 3:** Comparison of basic characteristics according to HBeAg status of women.

**Characteristics**	**HBeAg positive**	**HBeAg negative**	***P*-value**
No. of participants	69	209	
Age at birth (years)	28.75 ± 3.42	30.57 ± 3.91	0.001
Education level			0.431
Junior middle school or less	5 (7.2)	27 (12.9)	
Senior middle school	15 (21.7)	580 (21.5)	
College or university	49 (71.0)	2,699 (65.6)	
Pre-pregnancy BMI			0.401
< 18.5	11 (15.9)	48 (23.2)	
18.5~23.9	53 (76.8)	142 (68.6)	
>24	5 (7.2)	17 (8.2)	
Primipara	36 (52.2)	131 (62.7)	0.122
Gestational diabetes mellitus	13 (20.0)	37 (18.9)	0.842
Placental abruption	9 (13.0)	31 (14.8)	0.714
Postpartum hemorrhage	1 (1.4)	9 (4.3)	0.464
Cesarean delivery	23 (33.8)	71 (34.5)	0.923
Male infant	28 (40.6)	100 (47.8)	0.294
Preterm labor	1 (1.4)	16 (7.7)	0.115
Infant illness after childbirth	4 (6.3)	17 (9.4)	0.434

BMI, body mass index; HBeAg, hepatitis B e antigen.

## 4 Discussion

To the best of our knowledge, this is the first study to assess the relationship between maternal HBV infection and PPD in a large Chinese population. Our study showed that 11.9% of the mothers were considered as PPD at 6 weeks postpartum. We did not observe a greater risk of PPD in mothers with HBV infection. In addition, HBeAg positivity did not increase PPD risk among mothers living with HBV. Remarkably, our study suggested that parity and postpartum hemorrhage were associated with the risk of PPD.

This study found that 11.9% of Chinese postpartum women had PPD, slightly lower than the results of a meta-analysis conducted by Nisar et al., which reported a PPD prevalence of 14.8% in women from mainland China (33). The possible reason is that the latter analysis included studies where participants had a past history of mental illness. In addition, Liu et al. conducted a cross-sectional study in Shanghai, China, and found that 23.2% of women had depressive symptoms at 6 weeks postpartum (34), which is approximately twice the proportion in our study. Possible explanations for the difference in the results includes the study design and sampling method. Their study was a cross-sectional study based on convenience sampling, higher participation rate of women with high-risk pregnancies or complications may contribute to a higher prevalence. Our study was a hospital-based cohort study, and excluded those mothers with stillbirth, birth defect, past psychiatric diseases, or family history of mental illness that may increase the risk of PPD, leading to a lower prevalence of PPD.

A cross-sectional study conducted by Liu et al. suggested that hepatitis B self-awareness was significantly associated with depression in a Chinese study of 500,000 adults (35). Additionally, several studies found that people with chronic hepatitis B had higher depressive scores (26, 27). However, a case-control study conducted by Arslan et al. showed no difference in depression and anxiety between HBV-infected children and healthy control children (36). Similarly, Gale's study did not find a significant association of hepatitis B and depression in adult population (37). Our study showed that maternal HBV infection did not increase the risk of PPD. Therefore, the association between HBV infection and depression differs greatly across studies. It is known that specific populations have higher rates of mental health disorders with internalized stigma contributing to this, such as those with HIV/AIDS (38). It is possible that internalized stigma, common in patients with HBV may also contribute to increased risk of depression (39). Several reasons may contribute to explain the result difference between our study and Liu' study. First, participants with higher education in our study accounted for 66.9%, which is much higher than the 5.9% reported by Liu et al. (35). The HBV infection rate of 7.3% in our study is also 2.3 times that of their study (3.2%). A recent review reported that a higher education level, having a family member with HBV infection, and knowing someone with HBV infection are associated with the reduced stigma (40). Second, Interferon, the common drug for the treatment of hepatitis B, has a side effect of depression (41). But it is a contraindication for pregnancy women to receive Interferon therapy. Third, free antenatal education was offered to expectant mothers in China, which protects the attenders from adverse mental health outcomes (42).

The positive status of HBeAg in the serum is considered a credible marker of viral replication, which is associated with a higher risk of HBV MTCT. Our study indicated that HBeAg positivity was not associated with PPD risk. Viral load and antiviral use during pregnancy were identified as strong predictors of HBV MTCT (43). However, our study lacks data on viral load and antiviral use, which prevents us assessing the association between HBV MTCT and PPD directly. Therefore, this may be an important area for future studies to evaluate.

Consistent with our study, previous study indicated that primiparity was a risk factor for PPD during the first month postpartum (44). The main reason is that primipara may have many concerns, such as baby care, crying, and feeding, which are strongly associated with depressive symptoms in the early postpartum period (45, 46). In addition, our study suggested that women with postpartum hemorrhage still had higher risk of PPD. Similar results could be found in the study by Parry-Smith et al. (47) and our previous research (48). Postpartum hemorrhage usually leads to an increase in hospital stay and comorbidities, which may exacerbate the psychological burden of women who have suffered from postpartum hemorrhage.

Our study has several strengths. First, the cohort study design that enabled us to assess HBV infection status before PPD screening, which contributed to understanding the association between HBV infection and PPD. Second, data from a large sample cohort were used, which yielded more convincing results. However, the following limitations should be considered. First, the assessment of PPD was based on a self-reported scale instead of a psychiatric examination, although the EPDS is a validated tool for screening PPD with high sensitivity and specificity. Second, some confounding variables that may contribute to the risk of PPD were not considered, such as social support (from a husband, parents, or friends). Third, our study excluded patients with past history of mental illness, family history, or infants with birth defects, which may underestimate the impacts of HBV infection on PPD. Fourth, one-fifth participants was excluded because some women receiving PPD screening did not have a prenatal examination in this hospital during pregnancy, resulting in missing data on HBsAg and HBeAg.

## 5 Conclusions

In summary, this study is the first to explore the relationship between maternal HBV infection and PPD, although HBV infection did not increase the risk of PPD in puerperium women with no psychiatric or family history. The absence of an association may be explained by the diversity of sociological, behavioral, and psychological factors that affect the mental health of puerperal women. However, there were a number of issues that would be worth addressing in future research, e.g., HBeAg positivity, prior history of mental health.

## Data availability statement

The raw data supporting the conclusions of this article will be made available by the authors, without undue reservation.

## Ethics statement

The studies involving humans were approved by Institutional Review Board of the Baoan Maternal and Child Health Hospital. The studies were conducted in accordance with the local legislation and institutional requirements. The participants provided their written informed consent to participate in this study.

## Author contributions

WH and XW did the statistical analysis and drafted the initial manuscript. ZY, YG, and XL contributed to assist with data collection and revised the manuscript. LM took part in the sample collection. SP contributed to the critical revision of the article. All authors contributed significantly to this work. All authors have contributed to and have approved the final manuscript.
