# Corrigendum: Investigating the relationship between hepatitis B virus infection and postpartum depression in Chinese women: a retrospective cohort study

**DOI:** 10.3389/fpubh.2023.1358590

**Published:** 2024-01-08

**Authors:** Wei Huang, Xiaoli Wu, Zhenzhen Yao, Yingping Gu, Xin Lai, Liping Meng, Songxu Peng

**Affiliations:** ^1^Department of Postpartum Rehabilitation, Maternal and Child Health Hospital of Hunan Province, Changsha, China; ^2^Center for Reproductive Medicine, Maternal and Child Health Hospital of Hunan Province, Changsha, China; ^3^Department of Maternal and Child Health, Xiangya School of Public Health, Central South University, Changsha, China; ^4^Department of Public Health, Baoan Maternal and Child Health Hospital, Jinan University, Shenzhen, China

**Keywords:** postpartum depression, HBV, obstetric factors, EPDS, PPD

In the published article, there was an error in the Funding statement. The correct Funding statement appears below.

